# Structural Alterations of the Temporomandibular Joint in Rheumatoid Arthritis: A Cone-Beam Computed Tomography Study

**DOI:** 10.3390/medicina62101866

**Published:** 2026-09-26

**Authors:** Malina Iris Maftei, Codrina Ancuţa, Ionuț Tărăboanță, Magda Antohe, Cristina Gena Dascalu, Cristina-Angela Ghiorghe, Ana-Maria Fătu, Oana Țănculescu, Cristina Iordache

**Affiliations:** Grigore T. Popa University of Medicine and Pharmacy, 16 Universității Street, 700115 Iași, Romania; maftei_iris_malina@d.umfiasi.ro (M.I.M.); codrina.ancuta@umfiasi.ro (C.A.); magda.antohe@umfiasi.ro (M.A.); cristina.dascalu@umfiasi.ro (C.G.D.); cristina.ghiorghe@umfiasi.ro (C.-A.G.); ana.fatu@umfiasi.ro (A.-M.F.); oana.tanculescu@umfiasi.ro (O.Ț.); marina.iordache@umfiasi.ro (C.I.)

**Keywords:** rheumatoid arthritis, temporomandibular joint, cone-beam computed tomography, temporomandibular disorders, condylar morphology

## Abstract

*Background and Objectives*: Rheumatoid arthritis (RA) may involve the temporomandibular joint (TMJ), but structural features distinguishing RA-related involvement from non-RA temporomandibular disorders (TMDs) remain incompletely defined. This study compared quantitative and qualitative cone-beam computed tomography (CBCT) findings between patients with RA and a symptomatic non-RA TMD comparison group and explored bilateral asymmetry and associations with RA-related characteristics. *Materials and Methods*: This single-center retrospective cross-sectional study included 84 adults (42 RA and 42 non-RA TMD). All participants had TMJ-related symptoms or signs and clinically indicated CBCT examinations available. Bilateral CBCT assessment included condylar and mandibular fossa morphometry, joint-space measurements, condylar position, and qualitative osseous abnormalities. Linear mixed-effects models and generalized estimating equations accounted for bilateral observations, with Holm correction for multiple testing. Exploratory clinical associations and intraobserver reliability were also evaluated. *Results*: Clinical TMJ manifestations did not differ significantly between groups. The inferential analyses showed lower condylar height and anterior joint space and greater condylar length, anteroposterior condylar length, and condylar-to-midsagittal distance in RA. The largest quantitative difference involved condylar height (−5.43 mm; adjusted *p* < 0.001). Cortical erosions (OR = 5.94; 95% CI, 2.57–13.70) and joint-space narrowing (OR = 3.96; 95% CI, 1.66–9.42) were more strongly associated with RA. Absolute bilateral asymmetry did not differ after multiplicity correction, although group-by-side interactions persisted for condylar height and condylar position. Longer disease duration correlated with greater condylar-height asymmetry (ρ = 0.62; adjusted *p* < 0.001). Intraobserver reliability was high to excellent. *Conclusions*: RA-related TMJ involvement appears to reflect combined condylar remodeling, vertical structural loss, altered condyle–fossa relationships, and erosive change rather than uniform degeneration. These findings support CBCT as a potentially useful tool for structural characterization, but prospective multimodal validation using observed patient-level data is required.

## 1. Introduction

Rheumatoid arthritis (RA) is a chronic, systemic autoimmune disease characterized by persistent synovial inflammation, progressive cartilage destruction, and subchondral bone erosion, ultimately leading to irreversible joint damage, functional impairment, and reduced quality of life [1]. The disease predominantly affects peripheral synovial joints and typically manifests as a bilateral and symmetrical inflammatory polyarthritis involving the small joints of the hands and feet. However, because RA is a systemic inflammatory disorder with a predilection for synovial tissues, virtually any synovial articulation may be involved during the disease [2].

The temporomandibular joint (TMJ) is a specialized synovial articulation that plays a fundamental role in mastication, speech, swallowing, and mandibular mobility [3]. Unlike most peripheral joints, the TMJ possesses unique anatomical and biomechanical characteristics, including fibrocartilaginous articular surfaces and an intra-articular disc that separates the joint into upper and lower compartments [4]. These structural features may influence both the pattern of inflammatory involvement and the clinical manifestations observed in patients with rheumatoid arthritis [5].

TMJ involvement in RA has been increasingly recognized, although its reported prevalence varies considerably among studies, ranging from approximately 4% to 86% [6]. Such variability may be attributed to differences in patient populations, disease duration, diagnostic criteria, and methods used for clinical and imaging assessment. Although TMJ manifestations can occur at any stage of the disease, they are more frequently observed in patients with longstanding RA and advanced structural damage [7]. Furthermore, the TMJ is rarely the first joint affected and may remain clinically silent for extended periods despite the presence of structural abnormalities.

Clinical manifestations of TMJ involvement are often nonspecific and may overlap with those encountered in temporomandibular disorders of non-inflammatory origin [8]. Patients may present with preauricular pain, facial pain, headaches, tenderness of the masticatory muscles, morning stiffness, limitation of mandibular movements, joint sounds such as clicking or crepitus, impaired mastication, and reduced mouth opening [9]. In advanced cases, progressive condylar destruction may result in malocclusion, anterior open bite, mandibular retrusion, and significant impairment of oral function [8]. These manifestations may substantially affect quality of life and contribute to the overall burden of disease.

Several factors have been associated with an increased risk of temporomandibular joint involvement in rheumatoid arthritis, including longer disease duration, high disease activity, seropositivity for rheumatoid factor (RF) and anti-citrullinated protein antibodies (ACPA), erosive disease, and advanced functional impairment [10]. TMJ manifestations are more frequently observed in patients with longstanding and severe RA and are thought to reflect cumulative inflammatory joint damage and progressive structural deterioration [6].

Despite the relatively high prevalence of clinical and imaging abnormalities, temporomandibular joint involvement remains frequently underrecognized in routine clinical practice [11]. Symptoms may be mild, nonspecific, or attributed to concomitant disorders affecting the masticatory system, resulting in delayed diagnosis and treatment [8]. Moreover, structural joint alterations may already be present in the absence of significant clinical manifestations. Consequently, imaging evaluation plays a pivotal role in the early detection and characterization of TMJ abnormalities, facilitating timely diagnosis and a more comprehensive assessment of disease burden [4].

Because clinical findings do not always correlate with the severity of structural joint damage, imaging plays a crucial role in the assessment of TMJ involvement in rheumatoid arthritis [12]. Several imaging modalities are currently available, including panoramic radiography, computed tomography (CT), particularly cone-beam computed tomography (CBCT), dynamic magnetic resonance imaging (MRI), and ultrasonography (US) [11]. Panoramic radiography is commonly used as an initial imaging modality because of its accessibility, low radiation dose, and cost-effectiveness; however, its diagnostic accuracy is limited by image distortion, superimposition of adjacent structures, and reduced sensitivity for detecting early osseous changes [13].

Magnetic resonance imaging is considered the reference standard for evaluating soft-tissue structures of the TMJ, including the articular disc, synovial membrane, retrodiscal tissues, and joint effusion. In addition, MRI allows identification of active inflammatory changes such as synovitis and bone marrow edema [11].

Conventional CT provides excellent visualization of osseous structures and remains highly accurate for detecting cortical and subcortical abnormalities, although its routine use is limited by higher radiation exposure [7,11].

Cone-beam computed tomography has emerged as an important imaging modality for evaluating osseous TMJ abnormalities. Compared with conventional CT, CBCT provides high-resolution three-dimensional images with lower radiation exposure, shorter acquisition times, and lower costs. Furthermore, CBCT enables detailed visualization of cortical and trabecular bone structures, facilitating the detection of subtle abnormalities such as condylar flattening, cortical erosions, osteophyte formation, subchondral sclerosis, pseudocysts, and osseous remodeling [13]. These characteristics make CBCT particularly valuable for assessing structural damage associated with inflammatory arthropathies.

Recent deep learning approaches have enabled automated segmentation of the mandibular condyles and glenoid fossae on CBCT images, facilitating three-dimensional reconstruction and supporting quantitative assessment of TMJ morphology [14,15]. These developments extend the potential of CBCT beyond conventional linear measurements, although segmentation performance remains dependent on image quality and anatomical complexity [15].

Despite the growing use of CBCT in maxillofacial imaging, relatively few studies have comprehensively investigated TMJ alterations in patients with rheumatoid arthritis using this technique [11,13]. Moreover, the relationship between CBCT-detected structural abnormalities and clinical manifestations of temporomandibular dysfunction remains incompletely understood. Improved characterization of these associations may contribute to earlier recognition of TMJ involvement and facilitate a more comprehensive multidisciplinary management of patients with RA [9].

Accordingly, the primary objective of this study was to compare quantitative and qualitative CBCT characteristics of the temporomandibular joints between patients with rheumatoid arthritis and patients with TMD-related manifestations without rheumatoid arthritis. Secondary objectives were to evaluate bilateral TMJ asymmetry and to explore associations between RA-related clinical characteristics and CBCT findings. We hypothesized that patients with RA would exhibit distinct osseous and joint-space alterations compared with the non-RA TMD group.

## 2. Materials and Methods

This single-center, retrospective, cross-sectional comparative study was based on clinical and imaging records generated during routine clinical care between March 2025 and July 2026. Following completion of the study period, eligible records were retrospectively identified and reviewed for the purposes of the present analysis. No CBCT examination or additional clinical procedure was performed specifically for research purposes.

A total of 136 patient records were assessed for eligibility. Of these, 61 belonged to patients with rheumatoid arthritis and 75 to patients without rheumatoid arthritis. In the rheumatoid arthritis group, 19 records were excluded: 16 because the patients did not present the temporomandibular disorder-related symptoms or clinical signs required for inclusion, one because of a history of temporomandibular joint trauma, one because the cone-beam computed tomography examination was unsuitable for the predefined standardized measurements, and one because the patient was younger than 18 years.

In the group without rheumatoid arthritis, 33 records were excluded: 29 because the patients did not present the temporomandibular disorder-related symptoms or clinical signs required for inclusion, two because the cone-beam computed tomography examinations were unsuitable for standardized measurements, and two because of a history of temporomandibular joint trauma or previous temporomandibular joint surgery.

The final sample comprised 84 adult patients. The rheumatoid arthritis group included 42 patients with temporomandibular disorder-related symptoms or clinical signs and a diagnosis of rheumatoid arthritis established according to the 2010 American College of Rheumatology/European Alliance of Associations for Rheumatology classification criteria. The non-rheumatoid arthritis comparison group included 42 patients with temporomandibular disorder-related symptoms or clinical signs and no documented diagnosis or history of rheumatoid arthritis.

All included patients had undergone cone-beam computed tomography for the clinical investigation of temporomandibular joint symptoms; therefore, no imaging examination was performed exclusively for research purposes. Complete clinical and CBCT data were available for all included participants. Both groups were selected from the same clinical setting and study period and were evaluated using the same clinical and imaging protocol. No formal individual or frequency matching was performed. The comparability of the age and sex distributions was evaluated after participant selection.

The study was conducted in accordance with the Declaration of Helsinki and was approved by the Research Ethics Committee of the “Grigore T. Popa” University of Medicine and Pharmacy, Iași, Romania (approval no. 544/19 February 2025). Written informed consent was obtained from all participants for the use of their anonymized clinical and imaging data for research purposes. The study design and participant selection are presented in Figure 1.

### 2.1. Eligibility Criteria

#### 2.1.1. Inclusion Criteria

Adult patients aged 18 years or older were eligible for inclusion if they had been evaluated for temporomandibular joint-related symptoms or clinical signs and had undergone cone-beam computed tomography for the clinical investigation of these manifestations. At least one of the following manifestations was required: pain in the TMJ region, joint sounds, crepitus, or limitation of mandibular movements. Multiple manifestations could coexist in the same patient.

Only patients with complete clinical records and CBCT examinations suitable for the predefined standardized bilateral measurements were included.

Patients assigned to the rheumatoid arthritis group were required to have a diagnosis of rheumatoid arthritis established by a rheumatologist and to fulfill the 2010 American College of Rheumatology/European Alliance of Associations for Rheumatology classification criteria. No minimum rheumatoid arthritis disease duration was required for inclusion. Disease duration was extracted from the medical records and analyzed as a clinical characteristic of the RA group.

Patients assigned to the non-RA TMD comparison group were required to have no current or previous documented diagnosis of rheumatoid arthritis. They were evaluated for the same TMJ-related manifestations and underwent the same clinical and imaging assessment as patients in the RA group.

#### 2.1.2. Exclusion Criteria

Patients were excluded if they were younger than 18 years or did not present any of the TMJ-related symptoms or clinical signs required for inclusion. Additional exclusion criteria applied to both groups included a history of significant TMJ or craniofacial trauma, previous TMJ surgery, congenital TMJ or craniofacial malformations, septic arthritis of the TMJ, maxillofacial or TMJ tumors, and pre-existing structural TMJ conditions capable of independently altering joint morphology.

Patients with rheumatoid arthritis or other systemic inflammatory rheumatic diseases known to affect the TMJ were excluded from the non-RA TMD comparison group. Patients with concomitant systemic inflammatory rheumatic diseases other than rheumatoid arthritis were excluded from the RA group.

Records were also excluded when the CBCT examination did not permit standardized bilateral assessment because of inadequate image quality, limited field of view, positioning errors, imaging artifacts, or incomplete visualization of one or both temporomandibular joints.

The same predefined eligibility criteria were applied to both groups. The higher proportion of excluded records in the non-RA group primarily reflected the larger number of individuals who did not meet the required TMJ-related symptom/sign criterion, rather than differences in the screening procedure.

### 2.2. Clinical Assessment

Clinical information was extracted retrospectively from the medical and dental records of the included patients. All participants had been evaluated according to the same clinical protocol by a rheumatologist and a dental specialist.

#### 2.2.1. Rheumatologic Assessment

For patients in the rheumatoid arthritis group, the rheumatologic assessment included disease duration, age at disease onset, morning stiffness, the number of tender and swollen joints, erythrocyte sedimentation rate, C-reactive protein, rheumatoid factor, anti-cyclic citrullinated peptide antibodies, and antinuclear antibodies.

Disease activity was assessed using the Disease Activity Score in 28 joints. Functional status was classified according to the Steinbrocker functional classification.

RA erosive or non-erosive status, current antirheumatic treatment, including conventional synthetic DMARDs (methotrexate or non-methotrexate drugs such as sulfasalazine, leflunomide, hydroxychloroquine), biologic DMARDs (TNF inhibitors, tocilizumab, rituximab), targeted synthetic DMARDs (JAK inhibitors) and systemic glucocorticoids were also extracted from the medical records and used for descriptive characterization of the RA cohort. Where available, treatment duration and stability of therapy at the time of CBCT were also recorded.

#### 2.2.2. Temporomandibular Joint Assessment

Clinical TMJ assessment followed the RDC/TMD Axis I examination framework and was performed by the same dental specialist for all participants. The framework was used to assess and record TMJ-related symptoms and clinical signs. The examination included a structured history and inspection and palpation of the TMJs and masticatory muscles. The clinical TMD examiner was blinded to participants’ RA status.

The following TMJ-related manifestations were recorded: pain in the TMJ region, joint sounds, crepitus, and limitation of mandibular movements. Because more than one manifestation could be present in the same patient, these clinical categories were not mutually exclusive.

Mandibular mobility was evaluated by recording maximum mouth opening, right and left lateral excursions, and mandibular protrusion. Pain occurring at rest or during mandibular movements, tenderness on palpation, and joint sounds detected during opening, closing, and lateral mandibular movements were also documented. Mandibular mobility measurements recorded in the clinical files were extracted for analysis.

For the non-RA TMD group, individual RDC/TMD Axis I diagnostic categories were not recorded in the analytical dataset. The group was characterized by TMJ-related symptoms or signs and bilateral CBCT findings, with exclusion of RA and other systemic inflammatory rheumatic diseases. Qualitative CBCT abnormalities were recorded as present or absent, without standardized grading of degenerative severity.

#### 2.2.3. Oral and Occlusal Assessment

The intraoral examination included assessment of dental and periodontal status, oral mucosal and supporting bone structures, occlusion, and mandibular–cranial relationships.

The presence and distribution of edentulous areas were recorded together with their prosthetic status. Participants were classified according to the absence of edentulism, the presence of unreplaced edentulous spaces, adequate prosthetic rehabilitation, or inadequate prosthetic rehabilitation.

The occlusal assessment included Angle Class II or Class III relationships, anterior open bite, crossbite, and other clinically relevant occlusal alterations that could influence temporomandibular joint function. Prosthetic rehabilitation was assessed according to the type of restoration, including fixed, implant-supported, removable, or combined prostheses, and its clinical adequacy.

Dental, prosthetic, and occlusal variables were recorded as potential biomechanical factors that could influence TMJ morphology independently of rheumatoid arthritis and were considered in the descriptive comparison between groups.

### 2.3. Cone-Beam Computed Tomography Assessment

#### 2.3.1. Image Acquisition

All included patients underwent cone-beam computed tomography for the clinical investigation of temporomandibular joint-related symptoms. The examinations were performed at the same radiology center using a Green X 16 CBCT system (Vatech Co., Ltd., Hwaseong-si, Republic of Korea) and were analyzed with Ez3D-i software version 5.1.

The acquisition parameters were 92–94 kVp and 5–7 mA, with a field of view of 16 × 9 cm, a scan time of 11 s, an exposure time of 9 s, and an isotropic voxel size of 0.2 mm. The available acquisition-console screenshot displayed a dose–area product (DAP) of 1240.30 mGy·cm^2^.

CBCT volumes were reconstructed using the “High-Resolution Bone” reconstruction kernel. Images were evaluated using Ez3D-i software on a 27-inch IPS LED monitor (LG 27HJ713C-B.AEE, LG Electronics, Seoul, Republic of Korea), with a native resolution of 3840 × 2160 pixels (8 megapixels).

The monitor’s specifications include factory calibration to the DICOM Part 14 Grayscale Standard Display Function (GSDF), with a DICOM-calibrated luminance of 250 cd/m^2^, a typical luminance of 350 cd/m^2^, and a contrast ratio of 1000:1.

Patients were examined in a standing position, with the Frankfort horizontal plane parallel to the floor and the facial midline aligned with the unit’s vertical positioning laser. Head movement was restricted using a chin support and bilateral temple supports. Patients were instructed to close gently into maximum intercuspation, maintaining their habitual tooth contacts without guided mandibular manipulation, and to remain still throughout acquisition.

#### 2.3.2. Image Evaluation

CBCT examinations were evaluated by a specialist in radiology and medical imaging with experience in maxillofacial imaging. Both temporomandibular joints were assessed using the same standardized imaging protocol. The CBCT examiner was blinded to participants’ RA status and clinical assessment data. Quantitative measurements and qualitative CBCT findings were recorded separately for the right and left TMJs.

A single radiologist performed all assessments to maintain consistent application of the image-analysis protocol across the study sample. This design did not include an independent second assessment and therefore did not permit evaluation of interobserver agreement.

Measurements were performed separately on the right and left sides. The quantitative analysis included the following parameters:

- depth of the mandibular fossa (DMF), measured in the sagittal plane;
- condylar width (CoW), measured in the coronal plane;
- condylar length (CoL), measured in the sagittal plane;
- condylar height (CoH), measured in the sagittal plane;
- mediolateral width of the condyle (MWC), measured in the axial plane;
- anteroposterior length of the condyle (ALC), measured in the axial plane;
- superior joint space (SJS), measured in the sagittal plane;
- anterior joint space (AJS), measured in the sagittal plane;
- posterior joint space (PJS), measured in the sagittal plane;
- distance from the geometric center of the condylar process to the midsagittal plane (DGC).

All linear measurements were recorded in millimeters.

Condylar height (CoH) was measured from the most superior point of the condylar head to a reference line passing through the deepest point of the mandibular notch. This reference line was perpendicular to a tangent drawn along the posterior border of the mandibular ramus, and CoH was measured parallel to that tangent.

For DGC measurements, the midsagittal reference plane was defined as the plane passing through the anterior nasal spine (ANS), posterior nasal spine (PNS), and basion (Ba). The geometric center of the condylar process was operationally defined as the midpoint of the segment connecting the most medial and most lateral points of the condylar cortex on the axial section displaying its greatest mediolateral extent. DGC was measured as the perpendicular distance from this midpoint to the midsagittal reference plane.

Basion was visible and identifiable in all included CBCT volumes acquired with the 16 × 9 cm FOV, allowing its use in defining the ANS–PNS–Ba midsagittal reference plane for DGC measurements.

For each bilateral quantitative variable, the absolute difference between the right- and left-side measurements was calculated for each patient and used as an indicator of interarticular asymmetry:

Absolute asymmetry = |right-side value − left-side value|

The signed right–left difference was retained in the database to determine the direction of any observed asymmetry.

The anatomical landmarks, reference planes, and definitions of the quantitative CBCT measurements are detailed in Table S1.

#### 2.3.3. Qualitative CBCT Assessment

In addition to quantitative morphometric measurements, each TMJ was evaluated for structural osseous abnormalities on CBCT images. Qualitative findings were assessed separately for the right and left joints using multiplanar reconstructions and were recorded as present or absent.

The examiner was calibrated using the image analysis criteria described by Ahmad et al. [16].

The qualitative assessment included cortical erosions, joint-space narrowing, bone destruction, subchondral sclerosis, condylar deformity/flattening, subchondral cystic changes, and osteophyte formation. Cortical erosion was defined as a localized interruption or loss of continuity of the cortical articular surface, whereas bone destruction referred to more extensive loss of normal osseous architecture. Subchondral sclerosis was recorded when increased subcortical bone density was identified, and subchondral cystic change was defined as a well-demarcated radiolucent area within the subchondral bone. Condylar deformity/flattening was recorded when loss or alteration of the normal convex condylar contour was present. Osteophytes were defined as marginal osseous projections arising from the articular surface. Joint-space narrowing was recorded as a binary visual assessment of reduced interarticular space on multiplanar CBCT reconstructions. Classification relied on the examiner’s judgment rather than a predefined quantitative threshold or a validated CBCT grading scale.

All qualitative findings were evaluated bilaterally by the same experienced radiologist using the same image-analysis protocol applied to the quantitative measurements.

### 2.4. Statistical Analysis

Statistical analyses were performed using IBM SPSS Statistics, version 31.0 (IBM Corp., Armonk, NY, USA). Continuous variables were summarized as mean ± standard deviation or median [interquartile range], according to their distribution, and categorical variables as frequencies and percentages. Normality was assessed using the Shapiro–Wilk test together with visual inspection of Q–Q plots, while homogeneity of variances was evaluated using Levene’s test.

Between-group comparisons of demographic and clinical variables were performed using the independent-samples *t*-test, Welch’s *t*-test when equality of variances was not satisfied, or the Mann–Whitney *U* test for non-normally distributed variables. Categorical variables were compared using Pearson’s chi-square test or Fisher’s exact test, as appropriate.

Bilateral quantitative CBCT measurements were analyzed using linear mixed-effects models with participant included as a random intercept and study group, examined side, and the group × side interaction included as fixed effects. Models were adjusted for age and sex. Binary qualitative CBCT findings were analyzed using generalized estimating equations with a binomial distribution and participant-level clustering. Results are reported as effect estimates with 95% confidence intervals.

Absolute right–left differences were used to quantify the magnitude of bilateral asymmetry, whereas signed right–left differences were retained to characterize its direction. Exploratory associations between RA-related characteristics and quantitative CBCT outcomes were assessed using Spearman’s rank correlation, whereas associations with binary CBCT outcomes were examined using univariable logistic regression.

Intraobserver reliability was assessed in a randomly selected 25% subsample. Quantitative CBCT measurements were repeated by the same examiner after a three-week interval and evaluated using intraclass correlation coefficients based on a two-way mixed-effects model for absolute agreement and single measurements. During reassessment, examinations were reviewed in the same order as in the initial assessment, with the initial results concealed. The examiner remained blinded to RA status and clinical data. Agreement for binary qualitative findings was evaluated using Cohen’s κ coefficient [17].

Holm correction was applied separately to six families of tests: between-group comparisons for the 10 quantitative CBCT parameters; group × side interactions for these parameters; between-group comparisons for the seven qualitative findings; group × side interactions for these findings; the 10 comparisons of absolute bilateral asymmetry; and the six exploratory RA–CBCT associations. A Holm-adjusted *p*-value < 0.05 was considered statistically significant within each family.

Because of the retrospective design, all records meeting the eligibility criteria during the study period were included and no a priori sample-size calculation was performed. A sensitivity analysis was conducted using G*Power, version 3.1.9.7 (Heinrich-Heine-Universität Düsseldorf, Düsseldorf, Germany). With 84 participants equally allocated between the two groups, α = 0.05, and 80% power, the minimum detectable standardized between-group difference was Cohen’s *d* = 0.62. This sensitivity estimate pertains to the between-group comparison and does not quantify power for the group × side interactions or the exploratory associations within the RA group. The sensitivity calculation did not account for multiple-testing adjustment and should not be interpreted as evidence that the sample size was sufficient for the full set of analyses.

## 3. Results

### 3.1. Participant Characteristics and Clinical TMJ Findings

The final study sample comprised 84 participants, including 42 patients with rheumatoid arthritis and 42 patients with TMD-related manifestations without rheumatoid arthritis. Women represented 76.2% of the RA group and 64.3% of the non-RA TMD group, with no significant difference in sex distribution between groups (*p* = 0.233).

The mean age was 52.67 ± 11.01 years in the RA group and 50.45 ± 11.36 years in the non-RA TMD group, with no significant between-group difference (*p* = 0.367). Women represented 76.2% of the RA group and 64.3% of the non-RA TMD group, with no significant difference in sex distribution (*p* = 0.233).

TMJ pain was reported by 33 patients with RA (78.6%) and 35 non-RA participants (83.3%; *p* = 0.578). Joint sounds were recorded in 25 (59.5%) and 23 (54.8%) participants, respectively (*p* = 0.659), while crepitus was present in 15 (35.7%) and 13 (31.0%) participants (*p* = 0.643). Limitation of mandibular movements was more frequent in the RA group (33.3% vs. 19.0%), although the difference was not statistically significant (*p* = 0.136). Participant characteristics are summarized in Table 1, while the RA-specific characteristics are presented in Table 2.

### 3.2. Quantitative CBCT Findings

Quantitative CBCT measurements obtained in the analysis are summarized in Table 3. Significant overall between-group differences were identified for condylar length (CoL), condylar height (CoH), anteroposterior condylar length (ALC), anterior joint space (AJS), and the distance from the geometric center of the condylar process to the midsagittal plane (DGC).

Compared with the non-RA TMD group, the RA group showed greater CoL (estimated marginal difference: 0.89 mm; 95% CI: 0.42 to 1.36; Holm-adjusted *p* = 0.003) and ALC (0.93 mm; 95% CI: 0.45 to 1.41; adjusted *p* = 0.002), whereas CoH was markedly lower (−5.43 mm; 95% CI: −6.46 to −4.41; adjusted *p* < 0.001). AJS was also lower in the RA group (−0.46 mm; 95% CI: −0.77 to −0.14; adjusted *p* = 0.033), while DGC was greater (1.68 mm; 95% CI: 0.66 to 2.69; adjusted *p* = 0.011).

No significant overall group effects were observed for mandibular fossa depth (DMF), coronal condylar width (CoW), mediolateral condylar width (MWC), superior joint space (SJS), or posterior joint space (PJS) after correction for multiple comparisons.

Significant group-by-side interactions were identified for CoH (adjusted *p* = 0.037) and DGC (adjusted *p* < 0.001), indicating that the right–left pattern of these measurements differed between the RA and non-RA TMD groups. No significant group-by-side interaction was retained for the remaining quantitative parameters after Holm correction.

### 3.3. Bilateral Asymmetry of Quantitative CBCT Measurements

The magnitude of bilateral asymmetry was evaluated using the absolute difference between right- and left-side measurements. Although greater absolute differences were observed in the RA group for DMF, ALC, SJS, AJS, PJS, and DGC, none of the between-group comparisons remained statistically significant after correction for multiple testing. The largest nominal between-group difference was observed for SJS, with a mean absolute right–left difference of 0.96 ± 0.87 mm in the RA group and 0.59 ± 0.30 mm in the non-RA TMD group (mean difference: 0.37 mm; 95% CI: 0.08 to 0.66; unadjusted *p* = 0.012; Holm-adjusted *p* = 0.125).

Overall, the magnitude of bilateral asymmetry did not differ significantly between the RA and non-RA TMD groups for any of the quantitative CBCT parameters after Holm correction. This finding should be distinguished from the group-by-side effects reported above, which evaluate directional right–left differences rather than the absolute magnitude of asymmetry. Between-group differences in absolute bilateral asymmetry are illustrated in Figure 2.

### 3.4. Qualitative CBCT Findings

Qualitative CBCT findings are summarized in Table 4.

Cortical erosions and joint-space narrowing showed the strongest associations with RA. Cortical erosions were identified in 66.7% of right and 71.4% of left TMJs in the RA group, compared with 26.2% and 28.6%, respectively, in the non-RA TMD group. After accounting for bilateral observations, RA was associated with higher odds of cortical erosions (OR = 5.94; 95% CI: 2.57–13.70; Holm-adjusted *p* < 0.001).

Joint-space narrowing was present in 78.6% of right and 95.2% of left TMJs in patients with RA, compared with 61.9% on both sides in the non-RA TMD group. The overall odds of joint-space narrowing were higher in the RA group (OR = 3.96; 95% CI: 1.66–9.42; Holm-adjusted *p* = 0.011).

Bone destruction, subchondral sclerosis, condylar deformity/flattening, subchondral cystic changes, and osteophytes were not significantly associated with RA after correction for multiple comparisons. No group × side interaction remained statistically significant for any qualitative CBCT finding after Holm correction.

Structural abnormalities were also frequent in the non-RA TMD group (Table 4). Condylar deformity/flattening was recorded in 36/42 right TMJs (85.7%) and 39/42 left TMJs (92.9%), while joint-space narrowing was present in 26/42 joints (61.9%) on each side. Right- and left-side frequencies were 13/42 (31.0%) and 15/42 (35.7%) for subchondral sclerosis, 22/42 (52.4%) and 19/42 (45.2%) for subchondral cystic changes, and 15/42 (35.7%) and 18/42 (42.9%) for bone destruction, respectively.

### 3.5. Associations Between Rheumatoid Arthritis Characteristics and CBCT Findings

Exploratory associations between RA-related characteristics and selected CBCT outcomes are presented in Table 5. Longer disease duration was associated with greater absolute right–left asymmetry in condylar height (Spearman’s ρ = 0.62; Holm-adjusted *p* < 0.001).

An inverse association was observed between disease duration and mean condylar height (ρ = −0.37; unadjusted *p* = 0.016), and higher DAS28 values were associated with lower mean anterior joint space (ρ = −0.35; unadjusted *p* = 0.022); however, neither association remained significant after correction for multiple comparisons. Similarly, the associations of disease duration with bilateral joint-space narrowing and of DAS28 with bilateral cortical erosions did not retain statistical significance after Holm adjustment.

Anti-CCP positivity was not significantly associated with bilateral cortical erosions. Thus, after multiplicity correction, disease duration was the only RA-related characteristic significantly associated with a quantitative measure of bilateral TMJ asymmetry. The associations between RA characteristics and selected CBCT findings are presented in Table 5.

Continuous associations were assessed using Spearman’s rank correlation. Binary outcomes were explored using univariable logistic regression. Odds ratios are presented with 95% confidence intervals. The Holm procedure was applied across the six prespecified exploratory analyses.

### 3.6. Measurement Reliability

Intraobserver reliability was high to excellent for all quantitative CBCT measurements, with intraclass correlation coefficients (ICCs) ranging from 0.88 to 0.97 (Table 6). The highest agreement was observed for condylar height (ICC = 0.97; 95% CI: 0.92–0.99), whereas anterior joint space showed the lowest, although still high, reproducibility (ICC = 0.88; 95% CI: 0.74–0.95).

Agreement for qualitative CBCT findings was substantial to almost perfect, with Cohen’s κ coefficients ranging from 0.78 to 0.92. Joint-space narrowing showed the highest agreement (κ = 0.92; 95% CI: 0.80–1.00), whereas cortical erosions showed the lowest agreement (κ = 0.78; 95% CI: 0.59–0.97). Overall, both quantitative and qualitative CBCT assessments demonstrated good intraobserver reproducibility. The intraobserver reliability of CBCT measurements is presented in Table 6.

## 4. Discussion

The current findings suggest a distinct morphometric and structural TMJ phenotype in patients with RA, when compared with a symptomatic non-RA TMD group. The main quantitative differences observed in the present study comprised lower CoH and AJS and higher CoL, ALC, and DGC, whereas DMF, CoW, MWC, SJS, and PJS showed no significant overall group effects after correction for multiple comparisons. This pattern argues against interpreting RA-related TMJ involvement as uniform condylar degeneration; instead, it may reflect a more complex alteration of condylar architecture and condyle–fossa relationships, in which vertical loss coexists with contour remodeling and positional changes. This interpretation is broadly consistent with the meta-analysis conducted by Campos et al. [1], which reported a greater frequency of osseous TMJ abnormalities in RA across the included CBCT studies, and with Semerci and Günen Yılmaz [2], who emphasized the value of CBCT for characterizing osseous involvement in rheumatic diseases. Wydra-Karbarz et al. [9] likewise highlighted the heterogeneous and frequently underrecognized nature of TMD in RA. The symptomatic comparator provides a clinically relevant context for these findings, but its diagnostic composition remains incompletely characterized. The frequent structural abnormalities documented in the non-RA group may have attenuated some between-group differences, although the extent of this influence cannot be determined. The observed differences should therefore be interpreted as associations within this symptomatic population, without assuming that they identify changes specific to RA.

The most important quantitative finding was the reduction in condylar height (CoH) in the RA group, with an estimated marginal difference of −5.43 mm (Holm-adjusted *p* < 0.001). Clinically, reduced CoH is consistent with cumulative osseous loss and vertical condylar remodeling, which in advanced disease may alter intermaxillary relationships, mandibular rotation, and joint function. This finding agrees with Mohamed et al. [8], who reported significantly lower condylar height in a CBCT study of 20 patients with RA and 20 healthy volunteers. Similar structural involvement was described by Bayar et al. [10] using HRCT, including abnormalities in condylar shape and height, condylar head resorption, and erosions, and by Kretapirom et al. [11], whose MRI-based study demonstrated more frequent and severe condylar osseous changes in RA than in non-RA TMD patients. Nevertheless, the magnitude of the CoH difference should not be directly compared across studies, because definitions of condylar height, multiplanar orientation, anatomical landmarks, and imaging modality vary substantially; even CBCT and MRI may yield systematic differences for the same measurement [17].

In contrast to the reduction in CoH, both CoL and ALC were approximately 0.9 mm greater in the RA group, suggesting that condylar involvement is not characterized by uniform dimensional loss. Vertical bone loss may coexist with flattening and anteroposterior remodeling, potentially increasing selected linear dimensions despite reduced condylar height. This interpretation is consistent with the high prevalence of condylar flattening reported by Rehan et al. [12] and Cordeiro et al. [13], affecting 89.3% and 78.7% of patients with RA, respectively. However, Mohamed et al. [8] did not identify a comparable overall increase in anteroposterior dimensions and reported inverse associations between disease activity/ACCP and selected condylar measurements. These differences may reflect the use of healthy controls in that study, whereas our comparator group comprised patients with TMD, as well as differences in measurement planes and anatomical landmarks. Because CBCT-derived condylar dimensions are sensitive to reconstruction orientation and baseline morphology, increased CoL and ALC should be interpreted as potential markers of geometric remodeling rather than condylar hypertrophy and should be validated using standardized three-dimensional analyses.

Reduced AJS (−0.46 mm) suggests a difference in the anterior condyle–fossa relationship, consistent with previous reports of joint-space reduction in RA [10] and imaging studies examining spatial TMJ relationships and condylar positional changes [2,18]. The lower AJS represents a smaller anterior distance between opposing osseous surfaces, whereas qualitative joint-space narrowing reflects an examiner-dependent visual assessment without a predefined quantitative threshold. Neither finding establishes cartilage loss or active synovitis. CBCT provides detailed information on osseous morphology and geometric joint relationships, while MRI is more informative for assessing synovial and other intra-articular soft-tissue abnormalities. These cross-sectional differences should therefore not be interpreted as evidence of progressive cartilage damage or ongoing inflammatory activity.

The greater DGC in the RA group (+1.68 mm) provides complementary information on transverse condylar position relative to the midsagittal plane. This geometric measure complements condylar dimensions and joint-space measurements but does not directly quantify displacement within the mandibular fossa. However, CBCT-derived joint spaces are static geometric measures, not direct indicators of cartilage thickness or synovial inflammation. The DGC values in the RA group require caution because it describes condylar position relative to the midsagittal plane, not directly to the mandibular fossa. Its limited standardization and potential dependence on mandibular dimensions and reconstruction orientation restrict interpretation. Without normalization to mandibular width, the DGC difference and its group-by-side interaction remain exploratory rather than independent evidence of altered condyle–fossa relationships. A normalized sensitivity analysis would help clarify whether these findings persist after accounting for mandibular size.

Among the qualitative findings, cortical erosions and joint-space narrowing showed the strongest associations with RA (OR = 5.94 and 3.96, respectively), both remaining significant after Holm correction. The higher frequencies of cortical erosions and visually assessed joint-space narrowing indicate differences in osseous findings and joint-space appearance between groups; however, the contribution of synovial inflammation or cartilage pathology cannot be determined from these CBCT findings alone [19]. Cortical erosion may be particularly useful in distinguishing RA-related involvement from nonspecific degenerative changes. Rehan et al. [12] reported erosions in 85.7% of patients with RA, while the meta-analysis by Campos et al. [1] confirmed higher frequencies of erosions and other osseous abnormalities in RA. Similarly, A study conducted by Kretapirom et al. [11] demonstrated more frequent and severe condylar bone changes in RA than in non-RA TMD patients, and another research conducted by Cordeiro et al. [13] identified degenerative osseous alterations in approximately 90% of their RA cohort. Cortical erosions and visually assessed joint-space narrowing were associated with RA in this sample; however, these associations do not establish diagnostic discrimination. Their clinical interpretation requires confirmation using explicit assessment criteria and independent observers.

The absence of significant between-group differences in subchondral sclerosis, condylar deformity/flattening, cystic changes, and osteophytes after Holm correction is also clinically informative. These features are common manifestations of TMJ remodeling and may occur in both inflammatory arthritis and non-RA degenerative TMD, limiting their specificity when a symptomatic TMD group is used as a comparator. Cordeiro et al. [13] reported condylar flattening in 78.7% and osteophytes in 39.3% of patients with RA, while Rehan et al. [12] found flattening, sclerosis, osteophytes, and subchondral cysts more frequently in RA than in controls. However, these studies employed different comparator populations. More comparable to the present design, Kretapirom et al. [11] showed that the severity of osseous abnormalities and synovial proliferation discriminated RA from TMD more effectively than common findings such as disc displacement. Earlier radiographic comparisons with osteoarthritis likewise suggested that condylar erosions are more characteristic of RA, whereas osteophytes and flattening are less disease-specific. Collectively, these findings support interpreting CBCT abnormalities as an integrated structural pattern rather than as isolated pathognomonic signs.

Asymmetry analysis adds an important aspect to the interpretation of RA-related TMJ involvement. Although several absolute right–left differences were numerically greater in the RA group, none remained significant after Holm correction, whereas directional group × side interactions persisted for CoH and DGC. These findings are not contradictory: absolute differences quantify the magnitude of asymmetry irrespective of direction, while interaction terms can identify distinct right–left patterns even when overall asymmetry is comparable between groups. Accordingly, the obtained results do not support generalized greater TMJ asymmetry in RA. Rehan et al. likewise found no significant side-specific CBCT differences and subsequently analyzed both joints together, consistent with the predominantly bilateral nature of RA [12]. Semerci et al. also reported bilateral MSCT abnormalities, although severity differed between sides [2], while Kretapirom et al. demonstrated substantial bilateral structural involvement without identifying linear asymmetry as a principal discriminator [11]. Thus, RA may primarily produce bilateral joint damage, with local occlusal, functional, muscular, or anatomical factors modulating side-to-side expression [19,20]. The CoH and DGC interactions indicate differences in the mean signed right–left contrast between the RA and symptomatic non-RA TMD groups. They do not, by themselves, establish an RA-specific asymmetry phenotype or greater absolute asymmetry in individual patients with RA. Their clinical significance and disease specificity require independent validation.

In the present study, the disease duration was the only RA-related variable that remained associated with absolute CoH asymmetry after Holm correction (ρ = 0.62). This finding may indicate that cumulative inflammatory and structural burden does not affect both TMJs at the same rate [21]. Previous studies are inconsistent: Kroese et al. suggested that pain and crepitus may occur early, whereas dysfunction and structural changes accumulate over time [22]. At the same time, another study conducted by Shahidi et al. found no significant association between RA duration and CBCT abnormalities [23]. Wydra-Karbarz et al. further emphasized the heterogeneity of this relationship in the context of modern antirheumatic therapy [9]. Accordingly, CoH asymmetry should be regarded as a potential marker of cumulative disease burden rather than evidence of duration-dependent unilateral resorption [24].

The associations of disease duration with mean CoH and bilateral joint-space narrowing, and of DAS28 with AJS and bilateral erosions, were nominally significant but did not survive multiplicity correction; anti-CCP positivity was likewise unrelated to erosions. This limited robustness is clinically plausible because CBCT reflects cumulative structural damage rather than systemic inflammatory activity at a single time point [25]. These measures capture different biological and temporal aspects of RA: current systemic disease activity and structural changes accumulated over time. Weak or nonsignificant DAS28–CBCT associations should therefore not be interpreted as evidence that inflammatory activity has no role in TMJ structural damage. The cross-sectional design cannot establish whether RA activity preceded or contributed to the observed abnormalities. Mohamed et al. reported associations between DAS28 and condylar dimensions and osteoarthritic changes, while anti-CCP correlated inversely with condylar height and anteroposterior size [8]. A previous study observed a trend between TMJ CT scores and DAS28 [26], and the results of other research linked TMJ involvement to DAS28 and peripheral radiographic damage, but not to CRP, ESR, or RF [27]. Conversely, Rehan et al. found no meaningful correlations between CBCT findings and clinical or laboratory markers [12]. Such discrepancies may reflect small cohorts, treatment effects, heterogeneous imaging definitions, and the fact that DAS28 does not assess the TMJ, a limitation also emphasized in recent ultrasonographic studies.

Antirheumatic treatment should also be considered when interpreting structural TMJ findings. Modern DMARD therapy may reduce inflammatory activity and limit subsequent structural progression; however, CBCT depicts cumulative osseous changes that may have developed during earlier periods of higher disease activity or different therapeutic exposure. Therefore, the structural abnormalities observed in this cross-sectional study cannot be attributed to current treatment status alone.

Regarding reliability, it suggests good-to-excellent intraobserver consistency, an essential requirement when interpreting submillimetric CBCT differences. This is consistent with Gorucu-Coskuner et al., who reported high reproducibility for linear TMJ measurements but lower agreement for morphological classifications, which may explain the lower κ for erosions than the ICC for CoH [17]. More recent three-dimensional protocols have achieved similarly high reliability for positional and morphologic assessment.

The significant imaging findings have potential functional implications. From a biomechanical perspective, lower CoH could be relevant to vertical mandibular support and occlusal relationships, while cortical erosions indicate compromised articular bone integrity. Greater CoL and ALC, reduced AJS, and visually assessed joint-space narrowing describe differences in condylar morphology and joint geometry whose relationships with mandibular movement and masticatory function warrant investigation. The clinical significance of the DGC difference remains uncertain.

These potential implications were not directly tested in the present study. Associations between the significant imaging parameters and pain severity, mandibular mobility, or masticatory disability were not established, and treatment response was not assessed. Rehan et al. likewise reported no correlation between CBCT abnormalities and clinical findings or TMD severity [12]. Accordingly, the observed structural differences should not be interpreted as predictors of clinical impairment or treatment requirements without further validation.

### 4.1. Limitations

Several limitations should be considered. The retrospective, cross-sectional, single-center design and the modest sample size (42 participants per group) restrict the precision of effect estimates, particularly for interaction terms and binary outcomes. The absence of individual TMD diagnostic categories and standardized degenerative severity grades prevented characterization of diagnostic subgroups within the non-RA comparator. This limits assessment of how the composition of that group influenced the findings, particularly for osteophytes, condylar flattening, and sclerosis. The study also does not permit comparisons with healthy individuals. CBCT provides detailed assessment of osseous morphology but cannot directly characterize synovitis, effusion, bone marrow edema, or disc pathology, for which MRI remains more informative, as emphasized by Kretapirom et al. [11] and Tenório et al. [21]. CBCT diagnostic performance is also influenced by imaging protocols and diagnostic criteria. Moreover, the use of a single observer for all quantitative and qualitative CBCT assessments is a further limitation. Repeated assessments demonstrated consistency within the same examiner, but agreement between independent observers was not evaluated. For cortical erosions, the intraobserver κ estimate of 0.78 (95% CI: 0.59–0.97) also indicates uncertainty in the degree of within-examiner agreement. Furthermore, joint-space narrowing was assessed visually without a predefined quantitative threshold or validated grading scale, making classification dependent on examiner judgment. These limitations warrant cautious interpretation of the associations of both findings with RA.

Although dental, prosthetic, and occlusal characteristics were documented in both groups, residual confounding by edentulism, bruxism, malocclusion, prosthetic status, and other biomechanical factors cannot be excluded. The modest sample size also limited the number of additional covariates that could be incorporated reliably into multivariable models. The sensitivity analysis indicated a minimum detectable standardized between-group difference of d = 0.62 at 80% power and α = 0.05, suggesting limited sensitivity to smaller differences under the assumptions of that calculation. Power for group × side interactions and exploratory RA–CBCT associations was not separately quantified and may have been limited, particularly for analyses restricted to the 42 participants with RA and after multiplicity correction. Nonsignificant findings therefore do not exclude clinically relevant interactions or associations and should not be interpreted as evidence of their absence.

The cross-sectional design precludes temporal or causal inference regarding the relationship between RA activity and TMJ structural changes. A single DAS28 assessment cannot characterize the preceding inflammatory burden that may have contributed to the abnormalities observed on CBCT.

In addition, regarding the image acquisition protocol, the 16 × 9 cm field of view reflected the clinical acquisition protocol used at the imaging center. Although it provided bilateral TMJ coverage, the study did not establish whether a smaller field of view could have provided equivalent diagnostic information at a lower radiation dose. This represents a limitation of the acquisition protocol.

Treatment exposure was heterogeneous with respect to drug class and duration. Although current therapy and, where available, treatment duration were documented, the study was not designed to determine treatment-specific effects on TMJ morphology. Consequently, residual confounding by previous antirheumatic therapy, treatment duration, and cumulative disease activity cannot be excluded.

DGC was analyzed as an absolute distance without normalization to mandibular width. Consequently, the analysis could not distinguish differences in relative condylar position from those associated with overall mandibular dimensions.

### 4.2. Future Directions

Future studies should integrate standardized three-dimensional and semi-automated methods capable of quantifying condylar volume, surface characteristics, and regional remodeling beyond conventional linear measurements. Patient-specific finite element analysis could further investigate how condylar height loss and erosive changes influence TMJ stress distribution under defined loading conditions. Building on the QCT-based modeling and digital image correlation validation approaches discussed by Monalisa et al. [28], future studies could combine individualized anatomy with calibrated bone material properties. Application to the TMJ would require joint-specific loading assumptions and experimental validation.

Larger multicenter cohorts would allow multivariable models incorporating age, sex, RA duration, DAS28, CRP/ESR, RF, anti-CCP status, DMARD or biologic therapy, dental status, and occlusal variables, while providing greater power for group × side interactions. Longitudinal studies are particularly needed to determine how structural TMJ changes evolve over time; however, repeated CBCT should remain clinically justified and follow dose-optimization principles.

## 5. Conclusions

In this retrospective, cross-sectional sample, patients with RA showed lower condylar height and anterior joint-space measurements, together with greater condylar length, axial anteroposterior dimensions, and DGC, compared with symptomatic non-RA participants. Cortical erosions and visually assessed joint-space narrowing were also associated with RA status.

No statistically significant between-group differences in absolute bilateral asymmetry were detected after correction for multiple comparisons. The CoH and DGC group × side interactions describe differences in directional right–left relationships and require further investigation. The association between disease duration and condylar-height asymmetry should likewise be considered exploratory.

These findings describe structural and geometric differences within the studied sample, without establishing causality or RA-specific diagnostic markers. Their clinical relevance requires confirmation in larger longitudinal studies using standardized imaging criteria and independent observers.

## Figures and Tables

**Figure 1:**
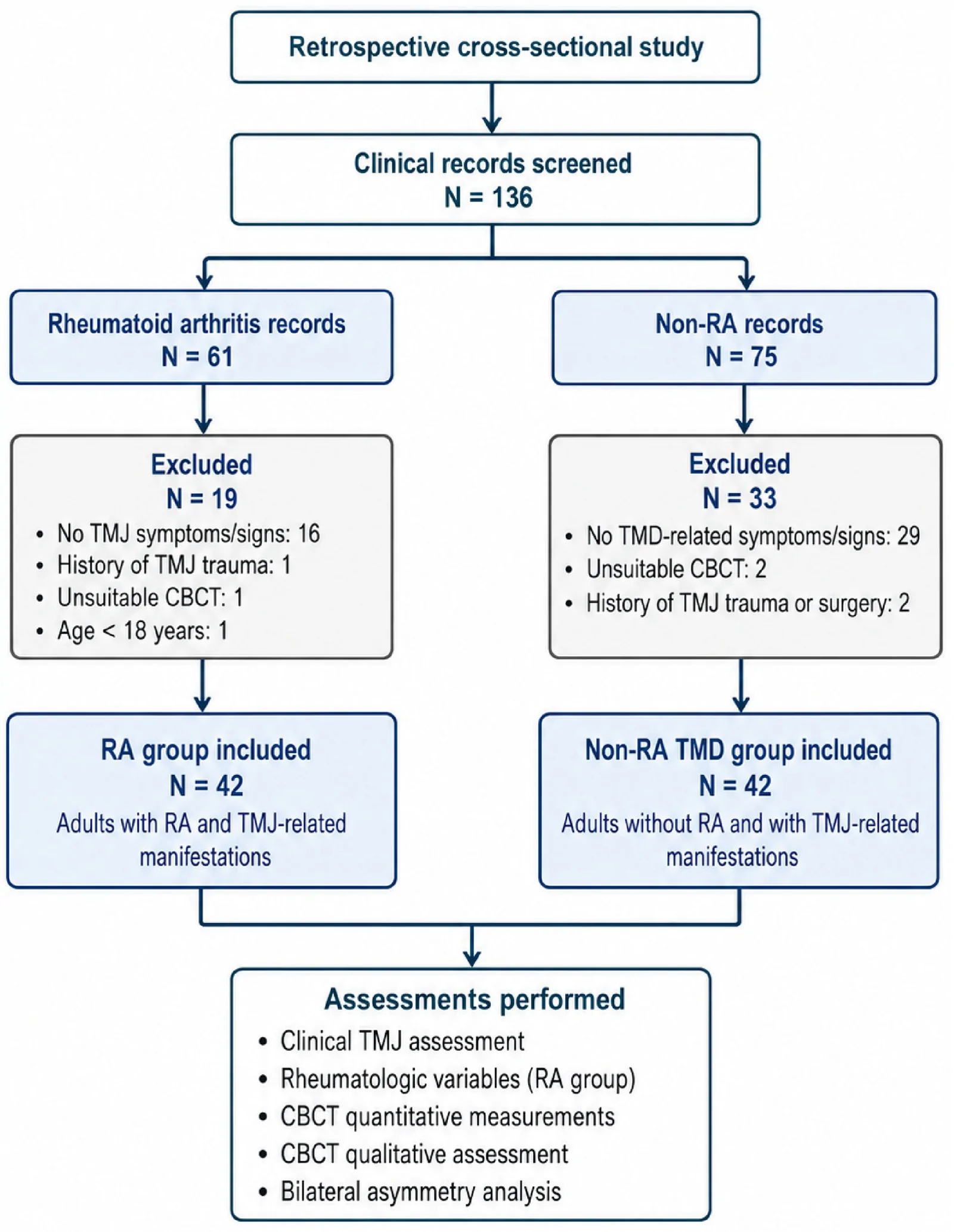
Study design and participant selection. Legend: RA, rheumatoid arthritis; TMD, temporomandibular disorders; TMJ, temporomandibular joint; CBCT, cone-beam computed tomography.

**Figure 2:**
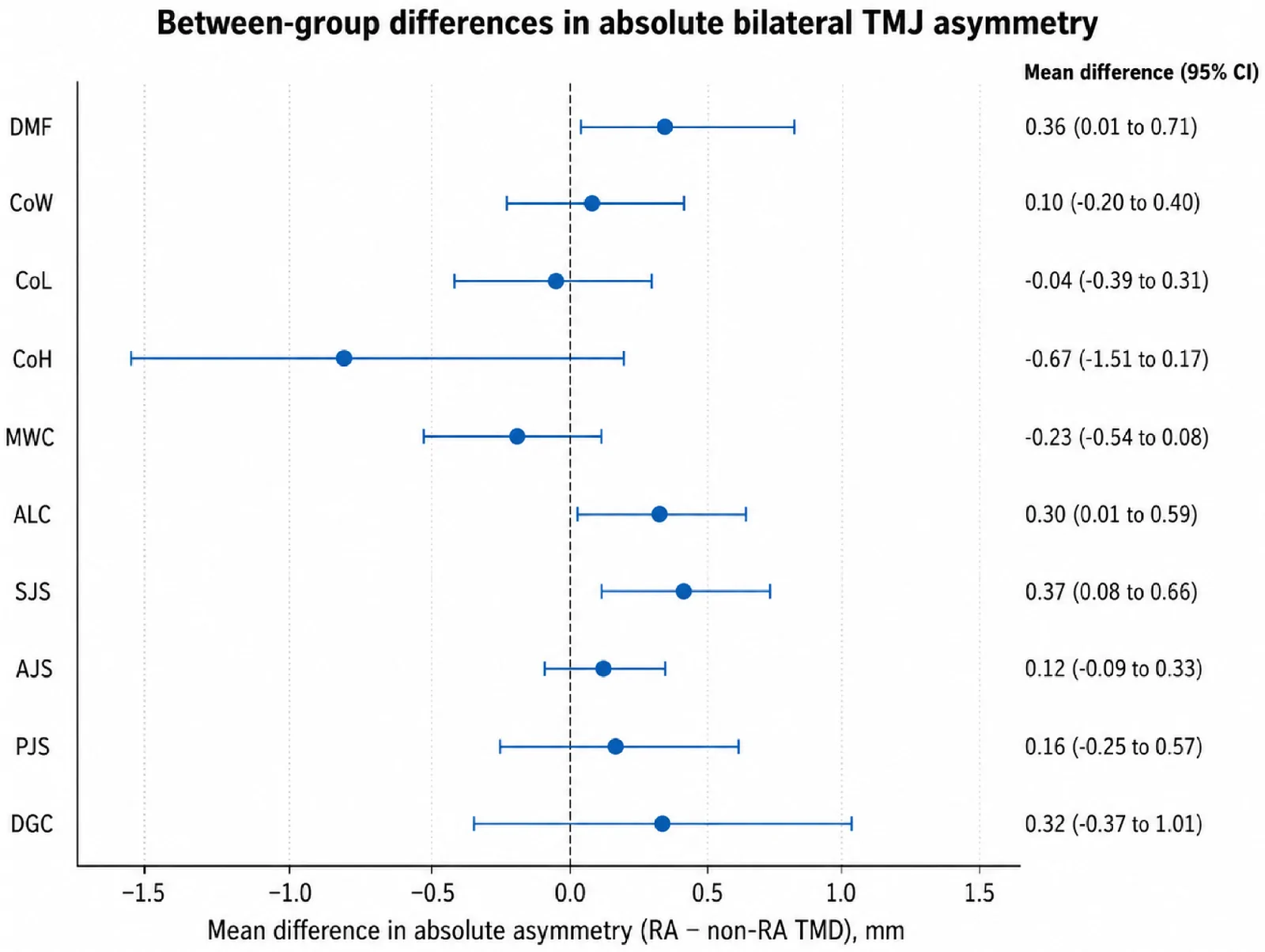
Between-group differences in absolute bilateral asymmetry.

**Table 1:** Demographic and clinical characteristics of the study population.

Characteristic	RA, *n* = 42	Non-RA TMD, *n* = 42	*p*-Value
Age, years	52.7 ± 11.0	50.5 ± 11.4	0.367
Female sex	32 (76.2)	27 (64.3)	0.233
TMJ pain	33 (78.6)	35 (83.3)	0.578
Joint sounds	25 (59.5)	23 (54.8)	0.659
Limitation of mandibular movements	14 (33.3)	8 (19.0)	0.136
Crepitus	15 (35.7)	13 (31.0)	0.643

**Table 2:** RA-specific characteristics.

Characteristic	RA Group (*n* = 42)
Disease duration, years	8.41 ± 4.18
DAS28	3.91 ± 1.10
ESR, mm/h	28.6 ± 15.4
CRP, mg/L	10.33 ± 8.15
Rheumatoid factor positive, *n* (%)	30 (71.4)
**Steinbrocker functional class, *n* (%)**	
Class I	10 (23.8)
Class II	18 (42.9)
Class III	11 (26.2)
Class IV	3 (7.1)
**Erosive status, *n* (%)**	
Erosive RA	24 (57.1)
Non-erosive RA	18 (42.9)
**csDMARD use, *n* (%)**	29 (69.0)
Methotrexate	21 (50.0)
Non-methotrexate csDMARDs	8 (19.0)
Sulfasalazine	2 (4.8)
Leflunomide	4 (9.5)
Hydroxychloroquine	2 (4.8)
**bDMARD use, *n* (%)**	17 (40.5)
TNF inhibitors	10 (23.8)
Tocilizumab	4 (9.5)
Rituximab	3 (7.1)
**JAK inhibitor use, *n* (%)**	5 (11.9)
**Glucocorticoid use, *n* (%)**	13 (31.0)

**Table 3:** Quantitative CBCT measurements and repeated-measures analysis.

Parameter	Non-RA TMD Right, Mean ± SD	RA Right, Mean ± SD	Non-RA TMD Left, Mean ± SD	RA Left, Mean ± SD	RA–Non-RA Marginal Difference Averaged Across Sides, mm (95% CI)	Holm-Adjusted *p*	Group × Side Effect, mm	Group × Side *p*
DMF	6.92 ± 0.94	7.12 ± 1.51	6.88 ± 0.79	6.89 ± 1.10	0.10 (−0.30 to 0.51)	1.000	0.19 (−0.34 to 0.72)	1.000
CoW	17.88 ± 2.17	18.32 ± 2.14	18.42 ± 2.08	18.41 ± 1.77	0.21 (−0.60 to 1.03)	1.000	0.45 (−0.23 to 1.13)	0.782
CoL	7.74 ± 0.87	8.64 ± 1.74	7.44 ± 1.13	8.32 ± 1.25	0.89 (0.42 to 1.36)	0.003	0.02 (−0.58 to 0.62)	1.000
CoH	26.35 ± 3.36	21.94 ± 2.67	27.18 ± 2.37	20.72 ± 2.89	−5.43 (−6.46 to −4.41)	<0.001	2.05 (0.67 to 3.43)	0.037
MWC	17.91 ± 2.12	18.59 ± 2.39	18.55 ± 2.03	18.66 ± 1.72	0.40 (−0.44 to 1.23)	1.000	0.57 (−0.13 to 1.27)	0.676
ALC	7.41 ± 1.02	8.39 ± 1.56	7.39 ± 0.98	8.27 ± 1.31	0.93 (0.45 to 1.41)	0.002	0.10 (−0.37 to 0.57)	1.000
SJS	3.40 ± 1.03	3.53 ± 1.37	3.04 ± 0.81	3.63 ± 1.54	0.36 (−0.12 to 0.84)	0.710	−0.46 (−0.89 to −0.03)	0.281
AJS	2.16 ± 1.02	1.92 ± 0.98	2.29 ± 0.50	1.62 ± 0.79	−0.46 (−0.77 to −0.14)	0.033	0.43 (0.06 to 0.80)	0.177
PJS	2.53 ± 1.22	2.33 ± 1.89	2.22 ± 0.87	2.43 ± 1.62	0.01 (−0.57 to 0.58)	1.000	−0.41 (−0.92 to 0.10)	0.676
DGC	49.66 ± 1.97	50.33 ± 2.60	49.14 ± 2.74	51.82 ± 2.88	1.68 (0.66 to 2.69)	0.011	−2.01 (−2.94 to −1.08)	<0.001

Marginal RA–non-RA differences represent model-estimated between-group contrasts averaged across the right and left sides and adjusted for age and sex; they are not side-specific estimates. The group × side interaction assesses whether the between-group difference varies by side.

**Table 4:** Qualitative CBCT findings.

CBCT Finding	Non-RA Right, *n* (%)	RA Right, *n* (%)	Non-RA Left, *n* (%)	RA Left, *n* (%)	OR (95% CI)	*p*adj	Group × Side *p*adj
Cortical erosions	11 (26.2)	28 (66.7)	12 (28.6)	30 (71.4)	5.94 (2.57–13.70)	<0.001	1.000
Joint-space narrowing	26 (61.9)	33 (78.6)	26 (61.9)	40 (95.2)	3.96 (1.66–9.42)	0.011	0.178
Bone destruction	15 (35.7)	10 (23.8)	18 (42.9)	20 (47.6)	0.90 (0.41–2.00)	1.000	0.393
Subchondral sclerosis	13 (31.0)	12 (28.6)	15 (35.7)	18 (42.9)	1.12 (0.53–2.35)	1.000	1.000
Condylar deformity/flattening	36 (85.7)	34 (81.0)	39 (92.9)	38 (90.5)	0.71 (0.26–1.99)	1.000	1.000
Subchondral cystic changes	22 (52.4)	14 (33.3)	19 (45.2)	19 (45.2)	0.68 (0.32–1.44)	1.000	0.393
Osteophytes	18 (42.9)	17 (40.5)	20 (47.6)	19 (45.2)	0.91 (0.42–1.97)	1.000	1.000

**Table 5:** Associations between RA characteristics and selected CBCT findings.

RA Characteristic	CBCT Outcome	Effect Estimate	95% CI	Unadjusted *p*	Holm-Adjusted *p*
Disease duration	Mean CoH	Spearman’s ρ = −0.37	—	0.016	0.078
Disease duration	Absolute CoH right–left difference	Spearman’s ρ = 0.62	—	<0.001	<0.001
DAS28	Mean AJS	Spearman’s ρ = −0.35	—	0.022	0.081
Disease duration	Bilateral joint-space narrowing	OR = 1.41 per year	1.00–1.97	0.048	0.096
DAS28	Bilateral cortical erosions	OR = 2.25 per unit	1.13–4.46	0.020	0.081
Anti-CCP positivity	Bilateral cortical erosions	OR = 1.91	0.45–8.04	0.378	0.378

**Table 6:** Intraobserver reliability of CBCT measurements.

CBCT Parameter	Reliability Statistic	Estimate	95% CI
DMF	ICC	0.94	0.86–0.98
CoW	ICC	0.95	0.88–0.98
CoL	ICC	0.92	0.82–0.97
CoH	ICC	0.97	0.92–0.99
MWC	ICC	0.95	0.88–0.98
ALC	ICC	0.93	0.84–0.97
SJS	ICC	0.90	0.78–0.96
AJS	ICC	0.88	0.74–0.95
PJS	ICC	0.89	0.76–0.95
DGC	ICC	0.96	0.90–0.99
Cortical erosions	Cohen’s κ	0.78	0.59–0.97
Joint-space narrowing	Cohen’s κ	0.92	0.80–1.00
Bone destruction	Cohen’s κ	0.84	0.67–1.00
Subchondral sclerosis	Cohen’s κ	0.82	0.63–1.00
Condylar deformity	Cohen’s κ	0.90	0.74–1.00
Subchondral cystic changes	Cohen’s κ	0.79	0.60–0.98
Osteophytes	Cohen’s κ	0.85	0.68–1.00

## Data Availability

The data presented in this study are available from the corresponding author upon reasonable request.

## Supplementary Materials

The following supporting information can be downloaded at: https://www.mdpi.com/article/10.3390/medicina62101866/s1, Table S1: Anatomical landmarks and operational definitions for linear CBCT measurements.

## Abbreviations

The following abbreviations are used in this manuscript:

ACPA	Anti-citrullinated protein antibodies
ACR	American College of Rheumatology
AJS	Anterior joint space
ALC	Anteroposterior length of the condyle
anti-CCP	Anti-cyclic citrullinated peptide antibodies
CBCT	Cone-beam computed tomography
CI	Confidence interval
CoH	Condylar height
CoL	Condylar length
CoW	Condylar width
CRP	C-reactive protein
CT	Computed tomography
DAS28	Disease Activity Score in 28 joints
DGC	Distance from the geometric center of the condylar process to the midsagittal plane
DMARD	Disease-modifying antirheumatic drug
DMF	Depth of the mandibular fossa
ESR	Erythrocyte sedimentation rate
EULAR	European Alliance of Associations for Rheumatology
HRCT	High-resolution computed tomography
ICC	Intraclass correlation coefficient
IQR	Interquartile range
MRI	Magnetic resonance imaging
MSCT	Multislice computed tomography
MWC	Mediolateral width of the condyle
OR	Odds ratio
PJS	Posterior joint space
RA	Rheumatoid arthritis
RDC/TMD	Research Diagnostic Criteria for Temporomandibular Disorders
RF	Rheumatoid factor
SD	Standard deviation
SJS	Superior joint space
TMD	Temporomandibular disorders
TMJ	Temporomandibular joint
US	Ultrasonography
