# Supplementary material for: Structural Alterations of the Temporomandibular Joint in Rheumatoid Arthritis: A Cone-Beam Computed Tomography Study
Source: Medicina (Kaunas). 2026 Sep 26;62(10):1866. doi: 10.3390/medicina62101866 (PMC13707342; doi:10.3390/medicina62101866)
Supplement: Supplementary file 1 [file medicina-62-01866-s001.zip › medicina-4540072-supplementary.pdf]

Table S1. Anatomical landmarks and operational definitions for linear CBCT measurements.

Parameter and reconstruction plane	Labeled points and anatomical locations	Operational measurement definition
DMF — Depth of the mandibular fossa; sagittal	E: most inferior point of the articular eminence. T: most inferior point of the postglenoid tubercle. F: point on the articular roof of the mandibular fossa with the greatest perpendicular distance from line E–T. F_0: perpendicular projection of F onto E–T.	Perpendicular distance F–F_0, measured from the articular roof to the reference line connecting E and T.
CoW — Condylar width; coronal	M□: most medial point of the condylar head cortex. L□:most lateral point of the condylar head cortex, identified on the same coronal section.	Linear distance M□–L□ on the coronal section displaying the greatest mediolateral extent of the condylar head.
CoL — Condylar length; sagittal	A_s: most anterior point of the condylar head cortex. P_s:most posterior point of the condylar head cortex, identified on the same sagittal section.	Linear distance A_s–P_s on the sagittal section passing through the operational condylar center C.
CoH — Condylar height; sagittal	S: most superior point of the condylar head cortex. N: deepest point of the mandibular sigmoid notch on the selected sagittal section. The posterior ramal tangent is drawn along the posterior border of the mandibular ramus. H: intersection of a line passing through S, parallel to this tangent, with a reference line passing through N, perpendicular to the tangent.	Linear distance S–H, measured parallel to the posterior ramal tangent and perpendicular to the reference line through N. H is a constructed reference point, not a point on the inferior condylar cortex.
MWC — Mediolateral width of the condyle; axial	M_a: most medial point of the condylar head cortex. L_a:most lateral point of the condylar head cortex on the same axial section.	Linear distance M_a–L_a on the axial section displaying the greatest mediolateral extent of the condylar head.

ALC — Anteroposterior length of the condyle; axial	A_a: most anterior point of the condylar head cortex. P_a:most posterior point of the condylar head cortex on the selected axial section.	Linear distance A_a–P_a, measured on the same axial section used for MWC.
SJS — Superior joint space; sagittal	S: most superior point of the condylar head cortex. F_s:intersection of a superiorly directed line through S, perpendicular to the axial reference plane, with the opposing articular cortex of the fossa roof.	Distance S–F_s along the defined superior–inferior direction.
AJS — Anterior joint space; sagittal	A_s: most anterior point of the condylar head cortex. E_a:nearest point to A_s on the opposing articular surface of the posterior slope of the articular eminence.	Shortest distance A_s–E_a between the designated condylar point and the opposing eminence surface.
PJS — Posterior joint space; sagittal	P_s: most posterior point of the condylar head cortex. F_p:nearest point to P_s on the opposing articular surface of the posterior wall of the mandibular fossa.	Shortest distance P_s–F_p between the designated condylar point and the opposing fossa surface.
DGC — Distance from the operational condylar center to the midsagittal plane; axial	C: midpoint of segment M_a–L_a on the axial section selected for MWC. MSP: plane defined by ANS, PNS, and Ba. C_0: orthogonal projection of C onto MSP.	Perpendicular distance C–C_0. C is an explicitly defined midpoint representing the operational condylar center; it is not a calculated volumetric centroid.
